# Polyphyllin II Triggers Pyroptosis in Hepatocellular Carcinoma via Modulation of the ROS/NLRP3/Caspase-1/GSDMD Axis

**DOI:** 10.3390/antiox15010075

**Published:** 2026-01-06

**Authors:** Huating Huang, Boran Ni, Qi Chen, Wenqi Wang, Zishuo Guo, Nan Wang, Rui Chen, Xingbin Yin, Changhai Qu, Jian Ni, Xiaoxv Dong

**Affiliations:** 1 School of Chinese Material Medica, Beijing University of Chinese Medicine, Beijing 102488, China; 20240941528@bucm.edu.cn (H.H.); 20250941611@bucm.edu.cn (Q.C.); 20240935097@bucm.edu.cn (W.W.); 20240941524@bucm.edu.cn (Z.G.); 20250935313@bucm.edu.cn (N.W.); 20250935194@bucm.edu.cn (R.C.); yxbtcm@163.com (X.Y.); quchanghai@bucm.edu.cn (C.Q.); 2 The Department of Endocrinology, Guang’anmen Hospital of China Academy of Chinese Medical Sciences, Beijing 100053, China; niboran@foxmail.com

**Keywords:** pyroptosis, ROS, calcium, Polyphyllin II, NLRP3, GSDMD

## Abstract

Pyroptosis is a type of programmed cell death (PCD) with pro-inflammatory properties, which is characterized by the swelling with bubbles and the release of LDH and inflammatory cell cytokines. Polyphyllin II (PPII) is the main active ingredient of the Chinese herb Rhizoma Paridis and has been proven to exert high efficacy against a variety of malignant tumors. At present, the anti-tumor research on PPII mainly focuses on apoptosis that is an anti-inflammatory type of PCD, but other potential modes of death cell death and mechanisms of PPII remain to be discovered. Here, we first found that PPII could effectively inhibit the growth of hepatocellular carcinoma (HCC) cells via pyroptosis. After treatment with PPII, the morphology of swelling with bubbles and the formation of pores in the cell membrane in HCC cells were observed, and LDH and cell cytokines (IL-1β, IL-18, IL-6, TNF-α, IFN-β, and IFN-γ) were released. Furthermore, the flow cytometry results showed that PPII could activate oxidative stress by increasing Ca^2+^ influx, thereby promoting the production of ROS to exert anti-tumor effects. RNA sequencing revealed that pyroptosis is closely linked to several signaling pathways, including the MAPK, TNF, Rap1, mTOR, and FoxO pathways, as well as the PD-L1 expression and PD-1 checkpoint pathway. An in vivo study demonstrated that PPII treatment suppressed liver tumor growth in mice by pyroptosis in a dose-dependent manner, and it showed no obvious side effects within a certain range. The Western blot results of tumor tissues revealed that the pyroptosis effect of PPII on liver cancer was associated with the activation of the NLRP3/Caspase1/GSDMD pathway, which upregulates the expression of NLRP3, Cleaved-Caspase 1, GSDMD-N, IL-1β, and IL-18 proteins and downregulates the expression of pro-Caspase 1 and GSDMD proteins. In summary, our findings revealed the pyroptosis effect and mechanism of PPII in HCC cells in vitro and in vivo, suggesting that PPII may be used as a potential pyroptosis inducer for HCC treatment in the future.

## 1. Introduction

Pyroptosis is a newly discovered type of programmed cell death (PCD) that is accompanied by an inflammatory response, which can induce immunogenic cell death (ICD) in tumor cells [1,2]. Different from other types of PCD such as apoptosis, pyroptosis is mainly carried out by the gasdermin (GSDM) family protein, which can be cleaved by functional proteins into gasdermin-N domain (GSDM-N) to perforate the plasma membrane [3]. This process further results in an osmotic pressure change, cell swelling and rupture, and the release of cytokines (such as IL-1β, IL-18, and HMGB1). Importantly, pyroptosis can not only kill tumor cells directly by osmotic lysis but also can release a variety of inflammatory mediators and cytoplasmic contents to increase the immunogenicity of tumors and enhance the immune effect of the body. It has been proved that inducing low levels of pyroptosis (less than 15%) can trigger highly effective anti-tumor immunity [4]. Recently, a growing number of studies have confirmed that a powerful immune stimulation triggered by pyroptosis or in combination with other therapies leads to an efficient and long-lasting anti-tumor response, supporting the metaphor that pyroptosis can “ignite a prairie fire with a single spark” [5,6,7,8]. For example, ZIF-8 nanoparticles can intrinsically induce pyroptosis by a Caspase-1/GSDMD-dependent pathway. The pyroptotic cell death releases massive amounts of inflammatory molecules to induce ICD for efficient in situ immunity initiation [7]. Therefore, pyroptosis-mediated cell death shows great potential in the field of tumor therapy.

The inflammatory Caspase family proteins are responsible for cleaving GDSM into the gasdermin-C domain (GSDM-C) and GSDM-N. Gasdermin D (GSDMD) is the direct substrate of Caspase 1/4/5/11, which can be divided into canonical inflammasome pathway dependent on Caspase-1 activation and non-canonical inflammasome pathway dependent on Caspase-4/5/11 activation [9]. In the classical pathway, the inflammasome NLRP3 plays a key role in the regulation of pyroptosis. Specifically, the inflammasome NLRP3 is activated by the stimulation of exogenous pathogens and endogenous danger signals, which further activates Caspase-1 downstream, thus initiating pyroptosis. In addition, the Caspase 3/GSDME pathway has also been shown to play an important role in the regulation of pyroptosis [10,11]. In recent years, pyroptosis mediated by the active components of traditional Chinese medicine (TCM) has gradually attracted attention in cancer research [12]. Cucurbitacin B (CuB), a tetracyclic triterpenoid compound, is the main active ingredient extracted from cucurbitaceae plants, and it has shown extensive anti-tumor activity in a variety of tumor cells, such as breast cancer, prostatic cancer, and lung cancer. Yuan et al. found that CuB could induce non-small-cell lung cancer cell pyroptosis in vitro and in vivo by triggering the NLRP3/GSDMD-dependent pathway [13]. Cinobufagin (CS-1), a main effective component of Chansu, has demonstrated significant anti-tumor efficacy in gastric cancer, colon cancer, etc. Ying et al. confirmed that CS-1 could effectively induce pyroptosis in MDA-MB-231 cells through the Caspase-3/GSDME signaling pathway, thus inhibiting tumor growth [14]. Mitochondrial dysfunction is involved in the process of cell death and is also the main mechanism of anti-tumor effects of many drugs. It is often accompanied by the massive production of ROS and the increase of Ca^2+^ influx, which plays an important role in activating NLPR3, regulating Caspase activity, and activating GDSMD protein in pyroptosis [15,16].

Polyphyllin II (PPII, Figure S1), a natural active steroidal saponin compound mainly extracted and isolated from the Chinese herb Rhizoma Paridis, has demonstrated potent anti-tumor efficacy against a range of malignancies, including liver cancer [17], lung cancer [18], breast cancer [19], ovarian cancer [20], and colorectal cancer [21]. The current evidence indicates that the anti-tumor mechanism of PPII is closely associated with the modulation of key signaling pathways such as PI3K/Akt, mTOR, and STAT3. To the best of our knowledge, current studies on the anti-tumor effects of PPII mainly focus on apoptosis, while other novel death modes and action targets in tumors have seldom been clarified. However, tumor cells killed by apoptosis lack sufficient immunogenicity to effectively activate the immune system, leading to the gradual development of resistance in tumor cells [22,23]. Therefore, the exploration of novel therapeutic methods for non-apoptotic cell death, especially cell pyroptosis, may provide a promising therapeutic strategy for cancer treatment.

**Scheme 1:**
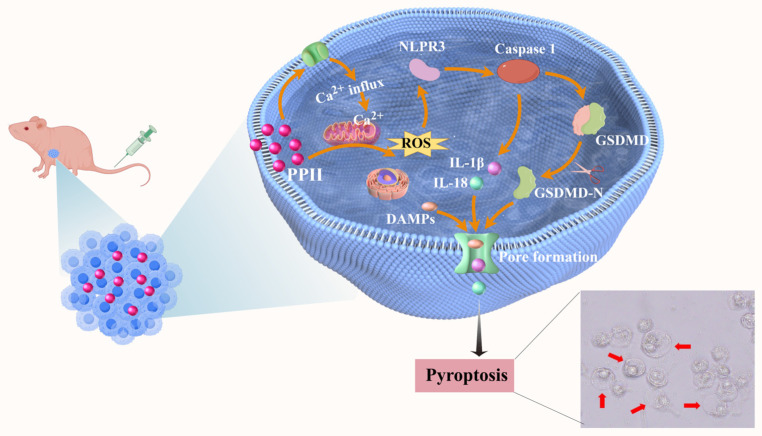
The schematic illustration of the mechanism of PPII inducing liver cancer pyroptosis through the NLRP3/Caspase 1/GSDMD signal pathway. Red arrows represent cell pyroptosis. Drawn with figdraw.

Based on the above facts, and taking the NLRP3/Caspase-1/GSDMD-dependent pyroptosis as the entry point, the anti-tumor activity of PPII in HCC cells was investigated. In this study, through a comprehensive series of in vitro and in vivo experiments, such as the cell morphology observation, lactic dehydrogenase (LDH) assay, flow cytometry analysis, enzyme-linked immunosorbent (ELISA) assay, and Western bolt analysis, we have confirmed the induction of pyroptosis by PPII for the first time. Specifically, PPII promotes oxidative stress by increasing Ca^2+^ influx, and then it produces ROS to induce pyroptosis in HCC cells, releasing LDH and pyroptosis-related cytokines IL-1β and IL-18. Moreover, the Western bolt results revealed that PPII induced pyroptosis via the modulation of the NLRP3/Caspase-1/GSDMD pathway (Scheme 1). These data provide support for PPII as an effective inducer of pyroptosis and offer an effective approach for a highly clinically efficient HCC treatment.

## 2. Materials and Methods

### 2.1. Materials

Polyphyllin II (purity ≥ 98%) was purchased from Shanghai Yuanye Biotechnology Co., Ltd. (Shanghai, China). Dulbecco’s modified eagle medium (DMEM), Roswell Park Memorial Institute 1640, PBS, penicillin–streptomycin (PS) solution, and 0.25% trypsin were acquired from Gibco (Grand Island, NY, USA). Fetal bovine serum (FBS) was purchased from Beijing Sijiqing Biotechnology Co., Ltd. (Beijing, China). The 2.5% glutaraldehyde fixing solution was purchased from Beijing Biorigin Biotechnology Co., Ltd. (Beijing, China). CCK-8 was purchased from Lableda Co., Ltd. (Beijing, China). Annexin V-FITC Apoptosis Detection Kit, LDH Cytotoxicity Assay Kit, ROS Assay Kit, and Fluo-4 Calcium Assay Kit were purchased from Beyotime Biotechnology (Shanghai, China). Human interleukin 18 (IL-18), human interleukin 1β (IL-1β), human interleukin 6 (IL-6), human interferon γ (IFN-γ), human tumor necrosis factor α (TNF-α), and human interferon β (IFN-β) ELISA kits were obtained from Shanghai Jianglai Biotechnology Co., Ltd. (Shanghai, China). Aspartate aminotransferase (AST), alanine aminotransferase (ALT), alkaline phosphatase (ALP), blood urea nitrogen (UREA), and creatinine (CREA) ELISA kits were purchased from Tianjin Hongke Biotechnology Co., Ltd. (Tianjin, China).

The antibodies anti-NLRP3 (ab263899), anti-Pro-Caspase1 (ab238972), anti-Cleaved-Caspase 1 (ab74279), anti-IL-1β (ab315084), anti-IL-18 (ab207324), and anti-β Actin (ab8227) were purchased from Abcam, and anti-GSDMD (EPR19829) and anti-GSDMD-N (EPR20829-408) were bought from Proteintech (Wuhan, China).

### 2.2. Cells and Animals

The liver cancer cell lines HepG2, Huh-7, and SNU-449 cells were purchased from Guangzhou Jenniobio Biotechnology Co., Ltd. (Guangzhou, China), Shanghai Jinyuan Biotechnology Co., Ltd. (Shanghai, China), Wuhan Pricella Biotechnology Co., Ltd. (Wuhan, China), respectively. The cells were cultured in DMEM or RPMI-1640 containing 10% FBS and 1% PS at 37 °C with 5% CO_2_. BALB/c male nude mice (5–6 weeks, 20 g) were purchased from Sipeifu Biological Technology Co. Ltd. (Beijing, China) and housed under a 12 h light/dark cycle with ad libitum access to food and water. All animal studies were performed according to the protocols approved by the Beijing University of Chinese Medicine Animal Welfare & Ethics Committee (Approval No.: BUCM-2023121106-4217; Approval Date: 26 February 2023) and complied with the Animal Research: Reporting of In Vivo Experiments (ARRIVE) guidelines.

### 2.3. Cell Viability Assay

HepG2, Huh-7, and SNU-449 cells were seeded in 96-well plates (5 × 10^3^ cells/well) overnight, and then they were treated with different concentrations of PPII (2, 4, 6, 8, 10, 15, and 20 μg/mL) for 24 h. Then, 10 μL of CCK8 solution was added to each well and incubated for 2 h. The absorbance at 450 nm was determined using a microplate analyzer (epoch2, BioTek Instruments, Winooski, VT, USA). Cell viability was calculated according to the following Formula (1), in which A_i_ means the absorbance of the treatment group, A_0_ means the absorbance of the blank medium, and A_c_ means the absorbance of the control group.

Cell viability rate (%) = (A_i_ − A_0_)/(A_c_ − A_0_) × 100% (1)

### 2.4. Apoptosis Assay

HepG2, Huh-7, and SNU-449 cells were cultured in 12-well plates (1.5 × 10^5^ cells/well) for 12 h, and then they were exposed to different concentrations of PPII (2, 4, and 6 μg/mL). After 24 h of treatment, cells were collected and washed twice with cold PBS. The obtained cells were stained in 295 μL binding buffer containing 5 μL Annexin V-FITC and 10 μL PI for 20 min in the dark. Flow cytometry was subsequently performed to detect the intracellular fluorescence using a BD FACSCanto II system (BD Biosciences, San Jose, CA, USA).

### 2.5. Cell Scratch Assay

HepG2, Huh-7, and SNU-449 cells were cultured in 6-well plates (2.5 × 10^5^ cells/well). After 12 h incubation, the cell monolayer was scratched using a 10 μL sterile pipette tip and then treated with different concentrations of PPII (2, 4, and 6 μg/mL). After 0 h, 24 h, and 48 h of scratching, the cell migration was observed under an inverted microscope. The scratch healing rate was calculated using the following Formula (2).

Scratch healing rate (%) = (0 h scratch width − 48 h scratch width)/0 h scratch width × 100%(2)

### 2.6. Observation of Cell Morphology

HepG2, Huh-7, and SNU-449 cells were inoculated in 12-well plates (1.5 × 10^5^ cells/well) and cultured for 12 h. Then, the cells were incubated with different concentrations of PPII (2, 4, and 6 μg/mL) for 12 h. The morphology of cells was observed under an inverted fluorescence microscope (ECLIPSE Ts2, NIKON Instrument, Tokyo, Japan).

### 2.7. Transmission Electron Microscopy

HepG2 cells were seeded in a 60 × 60 mm^2^ cell culture dish (7 × 10^5^ cells/dish) overnight, and then the different concentrations of PPII (2, 4, and 6 μg/mL) were added into the dish for 24 h of treatment. The cells were washed with PBS 2–3 times, fixed with 2.5% glutaraldehyde for 20 min, and collected. Then, they were fixed in 1% osmic acid solution for 1–2 h, dehydrated with a gradient-concentration ethanol solution, embedded in resin, subjected to gradient heating (35 °C~60 °C~80 °C) for 5 h at each step, and then sliced. The cells were observed with TEM (JEM-1200 EX, JEOL Instrument, Tokyo, Japan).

### 2.8. LDH Release Assay

HepG2 cells were seeded in 96-well plates (1 × 10^5^ cells/well) for 12 h of growth and then treated with PPII (2, 4, and 6 μg/mL) for 24 h. The supernatant of different groups was collected by centrifugation (400× *g*, 5 min), mixed with the LDH working solution, and then incubated at room temperature for 30 min. Finally, the absorbance at 490 nm was measured with a microplate analyzer.

### 2.9. ROS Assay

HepG2 cells were seeded in 12-well plates at a density of 1.5 × 10^5^ cells for overnight growth and incubated with different concentrations of PPII (2, 4, and 6 μg/mL) for 12 h. The cells were stained with the 0.5 mL DCFH-DA probe (10 μmol/L) at 37 °C for 20 min in a cell incubator and then observed using an inverted fluorescence microscope. At the same time, the cells were collected by centrifugation (500× *g*, 5 min) after being digested with 0.25% trypsin, and the intracellular fluorescence was quantitatively determined by flow cytometry (BD FACSCanto II, BD Biosciences, San Jose, CA, USA).

### 2.10. Ca^2+^ Assay

HepG2 cells (1 × 10^5^ cells/well) seeded in 12-well plates were treated with different concentrations of PPII (2, 4, and 6 μg/mL). After 12 h, the cells were collected by centrifugation (600× *g*, 5 min), washed once with PBS, and then stained with Fluo-4 (500 μL) at 37 °C for 30 min in the dark. The cells were suspended in 300 μL of detection buffer and analyzed by flow cytometry.

### 2.11. ELISA Assay

HepG2 cells were cultured in 12-well plates (1.5 × 10^5^ cells/well) for overnight growth and treated with different concentrations of PPII (2, 4, and 6 μg/mL). After 24 h, the supernatant was collected and centrifuged (1000× *g*, 10 min) to remove particles and polymers. The content of IL-18, IL-1β, IL-6, TNF-α, IFN-γ, and IFN-β in the supernatant was measured at 450 nm using a microplate analyzer according to the ELISA kit.

### 2.12. RNA Sequencing (RNA-Seq) Analysis

Total RNA was extracted from HepG2 cells using TRIzol^®^ Reagent according to the manufacturer’s instructions (Beyotime). High-quality RNA (OD 260/280 = 1.95, OD 260/230 = 2.12, RNA quality numbers (RQN) = 10.00 > 4.5) was used to construct an RNA-seq library (Illumina^®^ Stranded mRNA Prep, Ligation), which was prepared using the NavaSeq Reagent Kit (Illumina, Inc., San Diego, CA, USA). The data generated from the Illumina platform were used for bioinformatics analysis. All data analyses were performed using the Majorbio Cloud Platform (www.majorbio.com, accessed on 12 November 2025).

### 2.13. Tumor Xenograft Animal Experiments

A 100 μL volume of HepG2 cell suspension (1.0 × 10^7^ cell/mL) were injected into the armpits of Balb/c nude mice to establish HepG2-bearing nude mice models. The tumor volume was calculated according to Formula (3), which could be used in the experiment when the tumor volume increased to about 80 mm^3^. Nude mice were randomly divided into 4 groups (*n* = 4) including the control group (saline) and the PPII group (5, 7.5, 10 mg/kg). PPII was dissolved and diluted using saline, and 100 μL of this solution was intravenously injected into mice every 2 days for 12 days, while the mice in the control group were given 100 μL saline. The body weight and tumor volume of mice were recorded every day. After treatment, blood, tumor tissues, and major organ tissues were collected for further experiments.

V = 0.5 × L × W^2^ (3)

V: the tumor volume; L: the length of the tumor; W^2^: the short diameter of the tumor.

### 2.14. Hematoxylin and Eosin (H&E) Staining

The tumor and major organ tissues (heart, liver, spleen, lungs, and kidneys) harvested at the end of treatment were fixed with the 4% paraformaldehyde buffer and then eluted with gradient ethanol. Embedding and sectioning were then performed for H&E staining.

### 2.15. Blood Biochemical Index Detection

The mice’s whole blood was kept at room temperature for 2 h and centrifuged (1000 r/min, 20 min) to collect the serum. The content of AST, ALT, ALP, UREA, and CREA was detected according to the manufacturer’s instructions.

### 2.16. Western Blot Analysis of Tumor

The tumor tissues were lysed with protein lysate for 30 min, and then centrifuged at 4 °C (12,000× *g*, 30 min) to collect the supernatant. The protein in the supernatant was quantitatively analyzed with the BCA protein assay kit and then denatured by boiling for 5 min. The proteins were separated in the SDS-PAGE gel and then transferred to the PVDF membrane after 30 min of constant flow at 30 mV (4 °C). After blocking with 5% non-fat dry milk for 2 h, the PVDF membrane was incubated with the primary antibody for NLPR3, Pro-Caspase1, Cleaved-Caspase 1, GSDMD, GSDMD-N, IL-1β, and IL-18 at 4 °C overnight. On the next day, the blot was incubated with the corresponding fluorescent secondary antibody (1:8000) at room temperature for 1.5 h. The signal of the blot was acquired by the ECL detection system and quantified by ImageJ (v1.8.0).

### 2.17. Statistical Analysis

All experiments were performed with at least three biological independent replications. Data were analyzed using GraphPad Prism 8 and were expressed as means ± SD. One-way variance analysis (ANOVA) and Tukey’s test were used to assess the statistical differences for normally distributed data with homogeneous variance; otherwise, non-parametric tests were used. * *p* < 0.05, ** *p* < 0.01, and *** *p* < 0.001 were considered to be statistically significant.

## 3. Results

### 3.1. PPII Enhanced Cytotoxicity of HCC Cells

**Figure 1:**
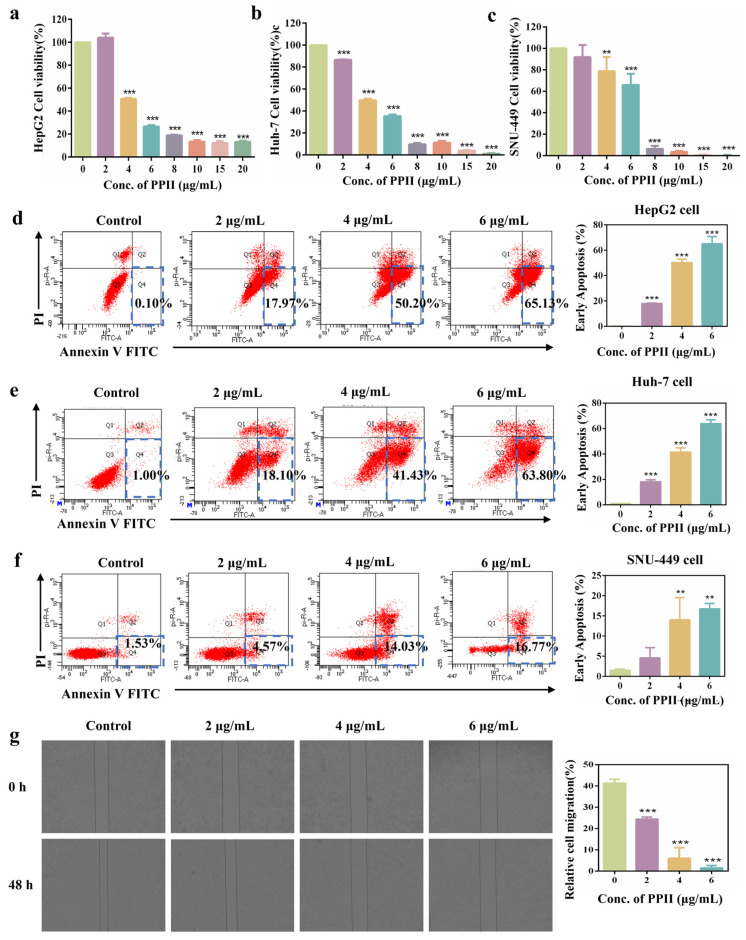
PPII enhanced the cytotoxicity of HCC cells. (**a**–**c**) The cell viability of HepG2, Huh-7, and SNU-449 cells treated with different concentrations of PPII for 24 h (*n* = 3). (**d**–**f**) Quantitative analysis of apoptosis rate in HepG2, Huh-7, and SNU-449 cells by flow cytometry with statistical evaluation (*n* = 3). (**g**) Representative wound healing images and statistical analysis of the migration rate at 48 h for HepG2 cells (*n* = 3). The *p* values were calculated by ANOVA with Tukey’s test. ** *p* < 0.01 and *** *p* < 0.001, compared with the control group.

Pyroptosis, an emerging inflammatory programmed cell death pathway, has been demonstrated to efficiently induce immunogenic cell death (ICD) in tumors compared to other pathways. This study aimed to explore the potential of PPII, an active component derived from traditional Chinese medicine, as a pyroptosis inducer in tumor cells. To evaluate the cytotoxicity of PPII on HCC, HepG2, Huh-7, and SNU-449 cells were treated with various concentrations of PPII, and the cell viability was measured using the Cell Counting Kit-8 (CCK-8) assay. As shown in Figure 1a–c, the survival rate of HepG2, Huh-7, and SNU-449 cells gradually decreased with increasing PPII concentrations, showing a significant dose dependence. PPII exhibited significant anti-proliferative effects against HepG2, Huh-7, and SNU-449 cells, with half inhibition rate (IC_50_) values of 4.44, 4.16, and 6.36 μg/mL, respectively. Therefore, 2, 4, and 6 μg/mL of PPII were selected for subsequent cell experiments. Flow cytometric analysis revealed that treatment with 6 μg/mL PPII significantly increased the apoptosis rate of HepG2 cells to 65.13 ± 5.65% (Figure 1d). Compared with the control group, PPII also significantly induced apoptosis in Huh-7 and SNU-449 cells (Figure 1e,f). Furthermore, the wound healing assay demonstrated that PPII markedly inhibited the migration ability of HepG2, Huh-7, and SNU-449 cells, which is a key indicator for suppressing tumor metastasis (Figure 1g and Figures S2–S4). Collectively, these data confirm the enhanced cytotoxicity of PPII on HCC cells.

### 3.2. PPII Induced HCC Cell Pyroptosis

**Figure 2:**
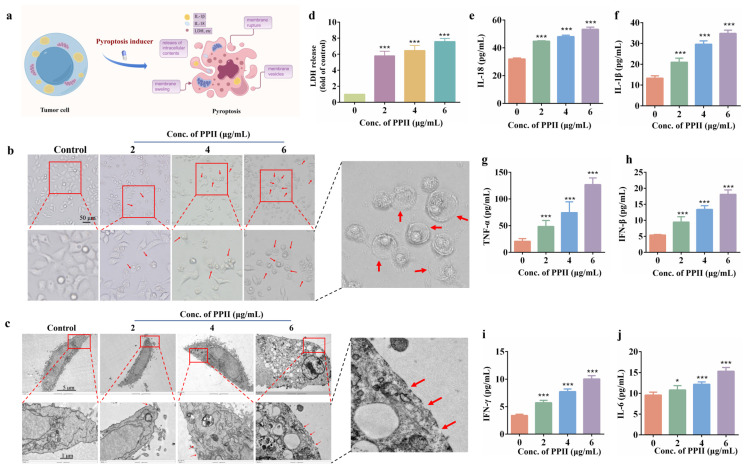
PPII induced pyroptosis in vitro. (**a**) The schematic diagram of the pyroptosis process in HepG2 cells. (**b**) The morphology changes in HepG2 cells after PPII treatment; red arrows represent cell pyroptosis. Scale bar = 50 μm. (**c**) The TEM image of HepG2 cells treated with PPII; red arrows indicate the missing part of cell membrane. Scale bar = 1 μm. (**d**) LDH release in HepG2 cells after treatment with different concentrations of PPII for 24 h (*n* = 3). *** *p* < 0.001, compared with the control group. (**e**–**j**) The levels of IL-18, IL-1β, TNF-α, IFN-β, IFN-γ, and IL-6 in the supernatant of HepG2 cells treated with different concentrations of PPII (*n* = 3). The *p* values were calculated by ANOVA with Tukey’s test. * *p* < 0.05, and *** *p* < 0.001, compared with the control group.

Pyroptosis and apoptosis are two kinds of programmed death modes of the body with some similar characteristics, such as DNA damage and nuclear coagulation, but they have different morphological characteristics. Different from apoptosis, pyroptosis is characterized by cell swelling, large bubble formation, and a balloon-like morphology [24], and is accompanied by the activation of the NLRP3 inflammasome and the release of Caspase-1-dependent cytokines IL-18 and IL-1β [25] (Figure 2a). The morphological observation of HepG2 cells showed cell swelling with bubbles, which revealed that cell death induced by PPII was a type of pyroptosis (Figure 2b). The same morphological features were also observed in Huh-7 and SNU-449 cells after PPII treatment (Figures S5 and S6). These changes are the characteristic morphological features of pyroptosis, which confirms that PPII can enhance pyroptosis in HCC cells. During pyroptosis, the activation of Caspase-1 leads to the formation of 10–18 nm pores in the cell membrane, resulting in the permeability of the plasma membrane and the release of LDH and inflammatory cytokines [26]. As shown by the red arrow in Figure 2c, the loss of cell membrane integrity on PPII-induced HepG2 was observed using TEM, which was another morphological feature of pyroptosis. LDH release assays were used to further evaluate the integrity of the cell membrane. Compared to the control group, PPII treatment significantly increased the LDH release of HepG2 cells in a dose-dependent manner (Figure 2d). The cytokines in the supernatant were determined by the ELISA assay. As displayed in Figure 2e,f, a 1.67-fold elevated level of IL-18 and a 2.61-fold elevated level of IL-1β, the two potent pro-inflammatory cytokines released during pyroptosis that can effectively recruit immune cells, were found in the PPII treatment group. Moreover, the content of TNF-α, IFN-β, IFN-γ, and IL-6 in the PPII group (6 μg/mL) was 6.10-fold, 3.34-fold, 2.95-fold, and 1.59-fold higher than that in the control group, respectively (Figure 2g–j). The release of these inflammatory cytokines is thought to play an important role in inducing ICD, which preliminarily indicates that pyroptosis may be potentially consistent with ICD-related signaling. Taken together, these results confirmed that PPII could induce pyroptosis in HCC cells and produce inflammation-related cytokines.

### 3.3. ROS Promote Pyroptosis in HepG2 Cells

**Figure 3:**
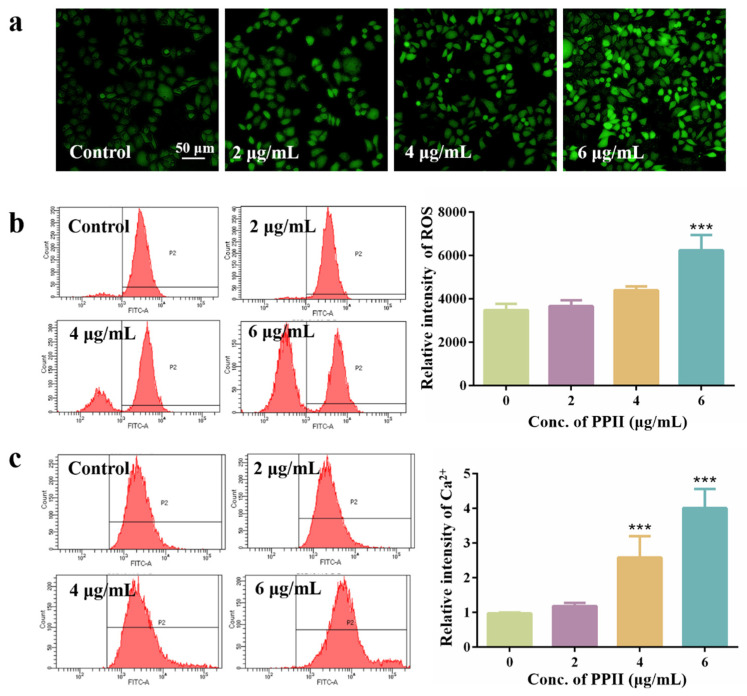
PPII induced pyroptosis associated with ROS. (**a**) Changes in intracellular ROS in HepG2 cells treated with different concentrations of PPII observed by fluorescence microscopy. Scale bar = 50 μm. (**b**) The flow cytometry analysis of intracellular ROS changes in HepG2 cells (*n* = 3). *** *p* < 0.001, compared with the control group. (**c**) The intracellular Ca^2+^ changes in HepG2 cells treated with different concentrations of PPII (*n* = 3). The *p* values were calculated by ANOVA with Tukey’s test. *** *p* < 0.001, compared with the control group.

ROS has been proved to play an important role in various death processes such as pyroptosis, apoptosis, PANoptosis, and ferroptosis, and is a key indicator for evaluating oxidative stress [27,28,29]. The DCFH-DA probe was used to detect ROS changes by an inverted fluorescence microscopy and flow cytometry. Compared with the control group, the green fluorescence of HepG2 cells was significantly enhanced in the PPII group (Figure 3a). The flow cytometry analysis results showed that the relative intensity of ROS in the PPII group (6 μg/mL) was 1.59-fold higher than that in the control group, which was consistent with the previous microscopic observation results, indicating that PPII-mediated pyroptosis was closely related to the generation of ROS (Figure 3b). The accumulation of Ca^2+^ can effectively inhibit tumor proliferation by activating oxidative stress, which acts as a crucial regulator of pyroptosis [30,31]. In this study, the level of Ca^2+^ in PPII-treated HepG2 cells was measured using the Fluo-4 fluorescent probe. As shown in Figure 3c, after treating with PPII for 12 h, the level of Ca^2+^ in HepG2 cells was significantly increased in a dose-dependent manner, suggesting that PPII could promote Ca^2+^ influx. These results indicated that PPII could increase Ca^2+^ accumulation and ROS production in HepG2 cells, thereby inducing cell pyroptosis.

### 3.4. Anti-Tumor Mechanism Analysis of PPII

**Figure 4:**
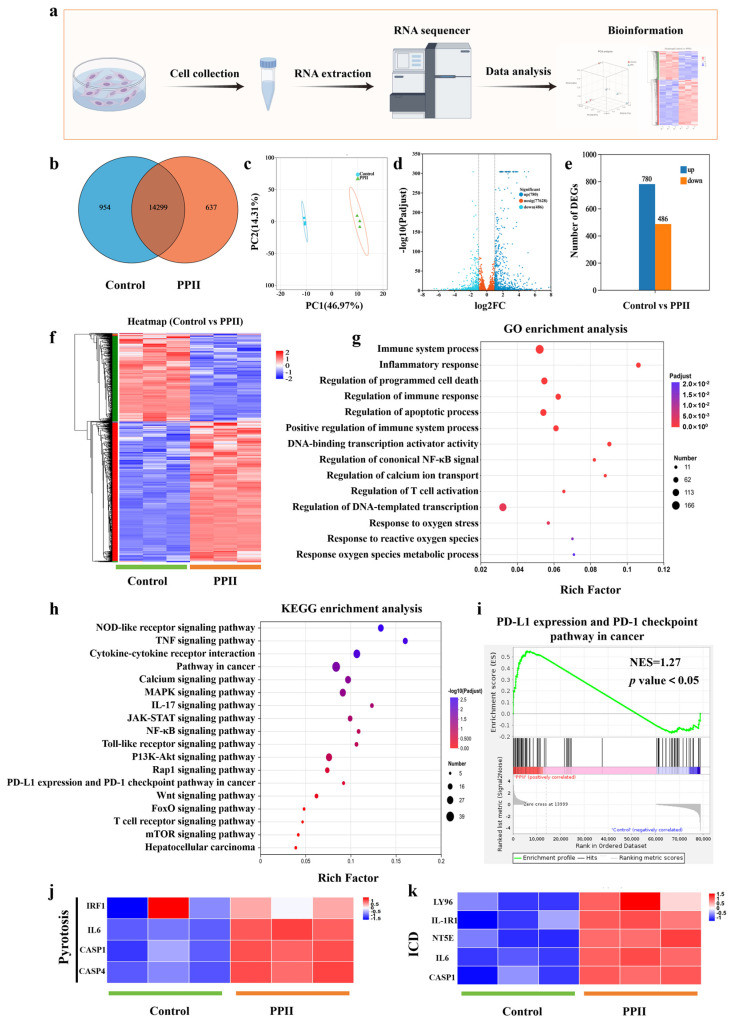
Therapeutic mechanisms of PPII treatment. (**a**) Schematic illustration of RNA-seq analysis of HepG2 cells. (**b**) Venn diagram of expressed genes in the PPII and control groups. (**c**) PCA of the PPII and control groups. (**d**–**f**) Volcano plot, bar plot, and heatmap of DEGs between the PPII and control groups. (**g**) GO enrichment analysis of DEGs. (**h**) KEGG enrichment analysis of DEGs. (**i**) GSEA of PD-L1 expression and PD-1 checkpoint pathway in cancer. (**j**,**k**) Heatmap of cluster analysis for representative genes involved in pyroptosis and ICD pathways.

To comprehensively evaluate the impact of PPII treatment on gene expression and transcription in HepG2 cells, we performed RNA-seq analysis on the PPII and control groups (Figure 4a). As shown in the Venn diagram, 14,299 genes were co-expressed in both groups, with 954 genes specifically expressed in the control group and 637 genes specifically expressed in the PPII group (Figure 4b). Principal component analysis (PCA) demonstrated good reproducibility among biological replicates and revealed significant differences in gene expression between the two groups (Figure 4c). The volcano plot and heatmap showed that, after PPII treatment, a total of 1266 differentially expressed genes (DEGs) were identified, including 780 upregulated and 486 downregulated DEGs (with an absolute fold change > 2 and a *p* value < 0.05) (Figure 4d–f). To further investigate the biological mechanism of PPII-induced cell death, we performed gene enrichment analysis using Gene Ontology (GO) and Kyoto Encyclopedia of Genes and Genomes (KEGG). GO enrichment analysis demonstrated that PPII treatment significantly promoted the expression of genes associated with response oxidative stress, the regulation of programmed cell death and immune response, etc. (Figure 4g). KEGG enrichment analysis further revealed differences in specific pathways. Immune-related pathways (such as the MAPK signaling pathway, TNF signaling pathway, Rap1 signaling pathway, PD-L1 expression, and PD-1 checkpoint pathway) and other pathways including the mTOR signaling pathway, FoxO signaling pathway, Wnt signaling pathway, and calcium signaling pathway were effectively activated (Figure 4h). It has been proved that the MAPK and TNF signaling pathways are involved in the mortal signaling of ROS [32]. Consistent with this, the DEGs in the PPII group were closely associated with the MAPK and TNF signaling pathways (Figure 4h). In addition, the KEGG analysis also highlighted the role of the FoxO signaling pathway, which is closely associated with ROS (Figure 4h). In this process, ROS regulates FoxO through protein interactions and post-translational modifications, whereas FoxO in turn controls the cellular levels of ROS [33]. It is known that PD-L1 plays a critical role in tumor immunity by binding to PD-1 on immune cells, thereby promoting tumor immune escape [34]. Therefore, blocking the PD-1/PD-L1 interaction has become a promising therapeutic strategy with considerable clinical potential. Based on KEGG pathway analysis, PPII modulates the PD-L1 expression and PD-1 checkpoint pathway in cancer (Figure 4h). Furthermore, Gene Set Enrichment Analysis (GSEA) indicated a positive correlation between the variations in gene expression and the PD-L1/PD-1 pathway (Figure 4i). As illustrated in Figure 4j,k, key genes associated with pyroptosis and ICD were significantly upregulated after PPII treatment, suggesting that PPII-induced pyroptosis may enhance anti-tumor immunity.

### 3.5. PPII Inhibited the Tumor Growth in Mice Models

**Figure 5:**
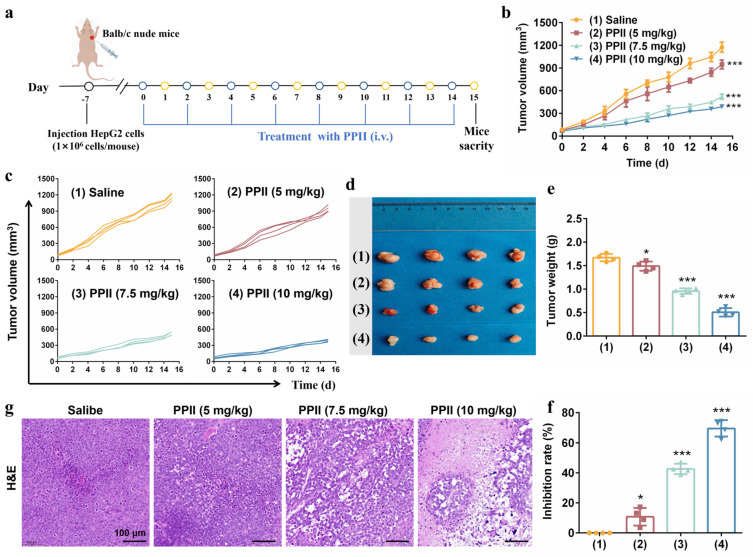
In vivo therapeutic effect of PPII. (**a**) Schematic illustration of the treatment plan on the HepG2-bearing male nude mice (*n* = 4). (**b**) Tumor volume–time curve of different treatment groups (*n* = 4). *** *p* < 0.001, compared with the saline group. (**c**) Tumor volume–time curve of each mouse in the saline group, PPII (5 mg/kg) group, PPII (7.5 mg/kg) group, and PPII (10 mg/kg) group (*n* = 4). (**d**) Photos of tumor tissues after different treatment groups for 14 days (*n* = 4). (**e**) Tumor weight of different treatment groups at 14 days (*n* = 4). * *p* < 0.05 and *** *p* < 0.001, compared with the saline group. (**f**) Inhibition rate of tumor weight of different treatment groups (*n* = 4). (**g**) H&E staining of tumor tissue of different treatment groups. Scale bars = 100 μm. The *p* values were calculated by ANOVA with Tukey’s test. * *p* < 0.05 and *** *p* < 0.001, compared with the saline group.

Inspired by the remarkable in vitro efficacy of PPII, we further evaluated the therapeutic effect of PPII in tumor-bearing nude mice. As noted, the nude mouse model, characterized by well-established tumorigenicity and immune-deficient nature, has been widely used for evaluating anti-tumor efficacy. When the tumor volume reached 80 mm^3^, the mice were divided into the control, PPII (5 mg/kg), PPII (7.5 mg/kg), and PPII (10 mg/kg) groups, and were treated according to the treatment plan in Figure 5a. Compared with the control group, the growth rate of tumor volume at the different concentrations of the PPII treatment groups slowed down by varying degrees. After 14 days of treatment, the tumor volumes of different PPII treatment groups (5, 7.5, and 10 mg/kg) were 947.64 ± 57.98 mm^3^, 520.14 ± 34.21 mm^3^, and 388.90 ± 23.05 mm^3^, respectively, which were significantly lower than that in the control group with 1175.35 ± 66.54 mm^3^ (Figure 5b,c). Solid tumors were removed and weighed at the end of treatment. As exhibited in Figure 5e,f, the tumor weight inhibition rate of the PPII group at 10 mg/kg was 69.61 ± 5.41% with a tumor weight of 0.51 ± 0.09 g, which was significantly higher than that in the PPII group at 7.5 mg/kg (0.96 ± 0.06 g), PPII group at 5.0 mg/kg (1.49 ± 0.10 g), and control group (1.67 ± 0.08 g), showing the most significant inhibitory effect (Inhibition rate (%) = (tumor weight of treatment group − tumor weight of control group)/tumor weight of control group × 100%). Despite the limited group size in this experiment (*n* = 4), the significant anti-tumor efficacy of PPII, in a dose-dependent manner, is clearly evidenced by the reductions in tumor volume and tumor weight and the increased tumor inhibition rate. Moreover, the H&E staining of the tumor tissue also confirmed the significant anti-tumor effects of PPII, which was manifested as the disappearance of tumor nucleus rupture, light cytoplasmic staining, and uniform staining of large necrotic areas (Figure 5g). In summary, the in vivo inhibition data support the conclusion that PPII exerts potent anti-tumor activity in tumor-bearing mice.

### 3.6. The In Vivo Biosafety of PPII

**Figure 6:**
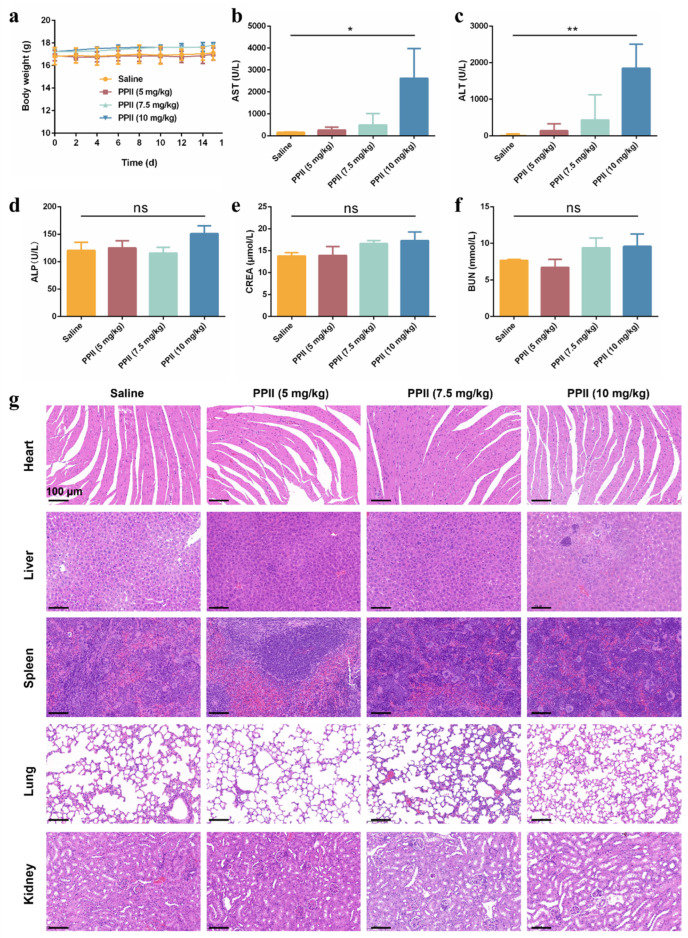
In vivo biosafety of PPII. (**a**) Body weight change curve of mice in different treatment groups (*n* = 4). (**b**–**f**) The levels of AST, ALT, ALP, CREA, and BUN in serum contents for different treatment groups (*n* = 3). (**g**) H&E staining of major organs (heart, liver, spleen, lungs, and kidneys). Scale bar = 100 um. The *p* values were calculated by ANOVA with Tukey’s test. * *p* < 0.05 and ** *p* < 0.01, compared with the saline group; ns indicates no significant differences.

Drug safety, along with efficacy, is a critical indicator for evaluating a treatment regimen. As shown in Figure 6a, the body weight of mice in the PPII treatment group did not decrease significantly, indicating that PPII had no obvious toxicity in vivo. The indexes of liver function (AST, ALT, and ALP) and kidney function (CREA and BUN) in serum were used to evaluate the potential liver and kidney damage of drugs. As presented in Figure 6b–f, the level of AST and ALT were increased in the high-dose PPII group (10 mg/kg) after 14 days of treatment, while there was no significant difference in ALP, CREA, and BUN, indicating that the dosage of PPII up to 10 mg/kg may have minor liver damage. Furthermore, the H&E staining results of liver sections in the 10 mg/kg PPII group showed a little inflammatory cell infiltration and liver nucleus rupture, whereas no significant damage was observed in other normal organs (Figure 6g). These results demonstrate that, while no significant hepatotoxicity was observed at doses below 10 mg/kg, mild liver injury occurred at 10 mg/kg, which might result from the accumulation of PPII in the liver, a digestive organ.

### 3.7. PPII Induced NLRP3/Caspase-1/GSDMD-Mediated Pyroptosis

**Figure 7:**
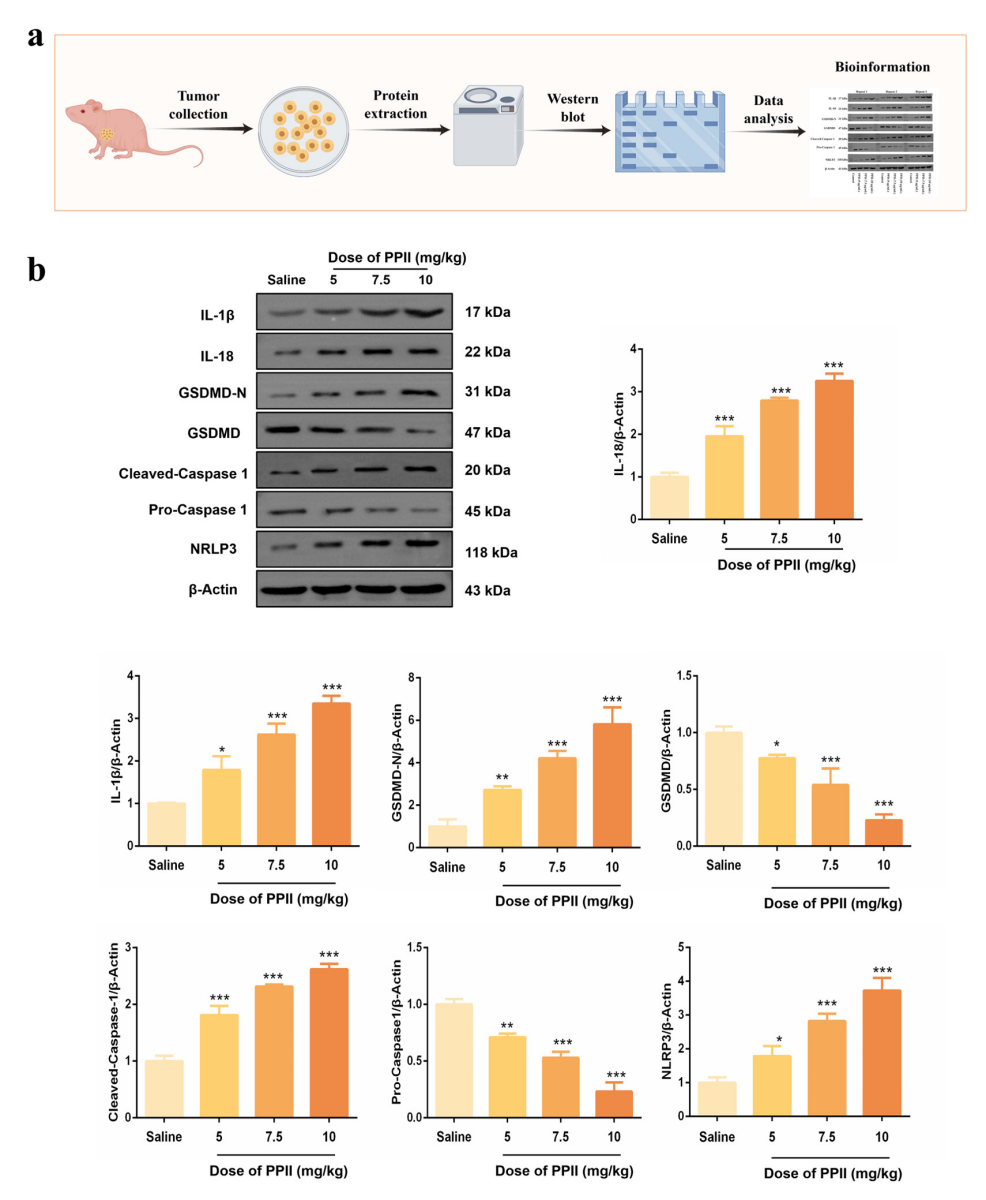
PPII induced pyroptosis by NLRP3/Caspase 1/GSDMD pathway. (**a**) Schematic illustration of Western blot for tumor tissues. (**b**) The expression and semi-quantitative analysis of IL-1β, IL-18, GSDMD-N, GSDMD, Cleaved-Caspase 1, Pro-Caspase 1, and NLRP3 proteins in tumor tissue (*n* = 3). The *p* values were calculated by ANOVA with Tukey’s test. * *p* < 0.05, ** *p* < 0.01, and *** *p* < 0.001, compared with the saline group.

NLRP3, a canonical inflammasome belonging to the Nod-like receptor family of pattern recognition receptors (PRRs), has been proved to play a key role in regulating cell pyroptosis [35]. It can be activated by a variety of stimulants, such as bacteria, viruses, ROS, ATP, and mitochondrial DAMPs. The activated NLRP3 can stimulate the activation of Caspase-1, which further cleaves GSDMD into the C-terminal domain of GSDMD (GSDMD-C) and the N-terminal domain of GSDMD (GSDMD-N). The GSDMD-N causes the formation of pores in the cell membrane by connecting with phosphatidylinositol, phosphatidic acid, and phosphatidylserine, leading to the maturation and release of IL-1β and IL-18 [1]. In this study, the underlying mechanism of PPII inducing pyroptosis in tumor tissues was investigated by the Western blot assay (Figure 7a). As shown in Figure 7b and Figure S7, the expression of NLRP3, Cleaved-Caspase 1, GSDMD-N, IL-1β, and IL-18 proteins significantly increased after PPII treatment, whereas the expression of Pro-Caspase 1 and GSDMD decreased. These data confirmed that PPII could induce pyroptosis in tumor cells via the NLRP3/Caspase 1/GSDMD pathway in tumor-bearing mice.

## 4. Discussion

Rhizoma Paridis, a rare traditional Chinese medicine, is the dried rhizome of *Paris polyphylla* Smith var. *yunnanensis* (Franch.) Hand.-Mazz or *Paris polyphylla* Smith var. *chinensis* (Franch.) Hara. It possesses many pharmacological activities such as anti-tumor, anti-inflammatory, antiviral, antibacterial, antioxidant, etc., showing a good application prospect. PPII, as the main component of Rhizoma Paridis, has been proved to have significant anti-tumor activity. In our previous study, we found that PPII can induce apoptosis through the mitochondrial pathway and death receptor pathway, thereby inhibiting HepG2 cells and HepG2 xenograft-bearing mice [17]. However, whether PPII can induce pyroptosis in HepG2 cells has not been reported. In recent years, polyphyllin I (PPI) and polyphyllin (PPVI), the other two critical components of Rhizoma Paridis, have been demonstrated to efficiently inhibit tumor growth by pyroptosis [36,37]. Encouraged by this, we hypothesized that PPII is expected to induce pyroptosis in HepG2 cells. Interestingly, we observed that the morphology of PPII-treated HepG2 cells exhibited swelling with bubbles (Figure 1d), which was preliminarily determined to exist with pyroptosis according to the literature [38]. Subsequently, in order to further verify the hypothesis, the cell membrane morphology observation, LDH release assessment, and ELISA assay were carried out. The results showed that, compared with the control group, the cell membrane was perforated, LDH was released in large quantities, and the secretion of pyroptosis cytokine (IL-1β and IL-18) was increased significantly after PPII treatment, which favorably confirmed the occurrence of cell pyroptosis. The Caspase family protein and ROS are widely reported to be pivotal in executing apoptosis and pyroptosis [7,39]. Combined with our previous study, PPII has been confirmed to induce HCC cell death via both pathways. However, the precise interplay and potential crosstalk between these mechanisms in our model remain to be fully elucidated. Future investigations employing specific inhibitors (e.g., NAC and Caspase-1 inhibitors) or evaluating common executioners and markers (e.g., Caspase-3, GSDME) will be crucial to dissect their individual contributions and synergistic relationships. Inflammatory cytokines, mainly secreted by immune cells, participate in and promote the body’s immune response. For example, IFN-γ is derived from activated T cells and NK cells, and it has been proved to play a key role in activating and strengthening adaptive tumor immunity mediated by T cells [40,41]. After PPII treatment, we observed a significant increase in the levels of inflammatory cytokines, including IL-6, TNF-α, IFN-β, and IFN-γ (Figure 2g–j). RNA-seq analysis also identified DEGs associated with ICD (Figure 4j,k). These results are consistent with the studies of other scholars [42,43], suggesting that pyroptosis may act as a potential inducer of tumor immunity. However, the present study primarily focused on confirming the occurrence of pyroptosis and its anti-tumor effects through a series of experimental approaches. As the current data are still insufficient to strongly support a direct link between pyroptosis and immune response, the potential of pyroptosis to induce ICD remains hypothetical. Therefore, further investigation into the robust ICD effects mediated by PPII-induced pyroptosis will be an important direction for our future research.

Biosafety is an important indicator for clinical evaluation of drugs. In this research, the biosafety of PPII was evaluated by weight change in mice, the biochemical analysis of blood, and the H&E staining of major organs. The body weight of mice did not decrease significantly during treatment, indicating that PPII had no obvious toxicity. Through blood biochemical indexes and H&E staining analysis, we found that PPII might cause mild damage to the liver when the concentration reaches 10 mg/kg. This result is similar to that of the study of Wang et al. They found that PPI caused minor damage to liver and kidney during treatment in HepG2 xenograft-bearing mice, in which the level of liver and kidney function (ALT, AST, ALP, and BUN) based on blood markers was elevated, and H&E staining showed some liver cell necrosis and glomerular atrophy [44]. Additionally, Jia et al. revealed that the crude extract of Rhizoma Paridis can induce a series of pathological reactions such as oxidative stress injury, inflammation, and apoptosis by interfering with the energy metabolism and lipid metabolism of the liver, eventually leading to liver injury [45]. These findings may be related to the fact that Rhizoma Paridis was a small toxic traditional Chinese medicine originally recorded in Sheng Nong’s herbal classic, and its therapeutic window was relatively narrow. This also suggests that we should pay attention to the therapeutic window period of PPII during treatment to reduce the occurrence of adverse reactions. Furthermore, the adverse reactions of PPII can also be ameliorated by nanotechnology, such as nanoparticles and liposomes. In our previous study, we effectively reduced the potential liver and kidney damage of PPII by delivering PPII to tumor tissues with PLGA, showing good biosafety [17,46].

In this research, through a series of in vivo and in vitro experiments, we confirmed that PPII could effectively inhibit tumor growth by inducing pyroptosis. Furthermore, we revealed that PPII induced pyroptosis via the modulation of the ROS/NLRP3/Caspase-1/GSDMD pathway. Inevitably, there are still some limitations that need to be addressed. While this study confirms the induction of pyroptosis, the underlying mechanisms require detailed characterization through subsequent rescue experiments. RNA-seq analysis has suggested potential pathways related to PPII-induced pyroptosis; however, definitive validation awaits further in vitro and in vivo investigations. In terms of the specific mechanism of PPII-induced pyroptosis, we only explored the classical pyroptosis pathway (Caspase 1/GSDMD pathway), while the Caspase 3/GSDME pathway has not been explored. In addition, due to the lack of reference clinical data, the dosage of PPII in animal experiments was mainly obtained through pre-experiments. Therefore, there are still many problems to be solved in order to truly achieve the clinical translation of PPII.

## 5. Conclusions

In summary, we demonstrated that PPII could significantly inhibit HCC growth through cell pyroptosis in vitro and in vivo for the first time. PPII induced cell pyroptosis by increasing Ca^2+^ accumulation and ROS production, leading to the release of LDH and inflammatory cell cytokines (IL-1β, IL-18, IL-6, TNF-α, IFN-β, and IFN-γ). The expression of related proteins in tumor tissues, including NLRP3, Pro-Caspase 1, Cleaved-Caspase 1, GSDMD, GSDMD-N, IL-1β, and IL-18, has an association with the activation of the NLRP3/Caspase-1/GSDMD pathway. This work will provide novel insights into the application of PPII-mediated pyroptosis for HCC treatment.

## Data Availability

The original contributions presented in this study are included in the article and Supplementary Materials. All sequencing data are available through the NCBl Short Read Archive (SRA, http://www.ncbi.nlm.nih.gov/sra/ accessed on 31 December 2025) under the accession number PRJNA1395326.

## Supplementary Materials

The following supporting information can be downloaded at: https://www.mdpi.com/article/10.3390/antiox15010075/s1, Figure S1. The chemical structure of PPII; Figures S2. Representative wound healing images of HepG2 cells at 24 h and 48 h (*n* = 3); Figures S3. Representative wound healing images of Huh-7 cells at different time points and statistical analysis of migration rates at 48 h (*n* = 3). The *p* values were calculated by ANOVA with Tukey’s test. *** *p* < 0.001, compared with control group; Figures S4. Representative wound healing images of SNU-449 cells at different time points and statistical analysis of migration rates at 48 h (*n* = 3). The *p* values were calculated by ANOVA with Tukey’s test. *** *p* < 0.001, compared with control group. Figure S5; The morphology changes of Huh-7 cells after PPII treatment for 24 h, red arrow represents cell pyroptosis. Scale bar = 200 μm; Figure S6. The morphology changes of SNU-449 cells after PPII treatment for 24 h, red arrow represents cell pyroptosis. Scale bar = 200 μm; Figure S7. Original bands of IL-1β, IL-18, GSDMD-N, GSDMD, Cleaved-Caspase 1, Pro-Caspase 1, and NLRP3 proteins in tumor tissue (*n* = 3).

## References

[1] Fang Y., Tian S., Pan Y., Li W., Wang Q., Tang Y., Yu T., Wu X., Shi Y., Ma P. (2020). Pyroptosis: A new frontier in cancer. Biomed. Pharmacother..

[2] Wang Y.Y., Li S.L., Zhang X.Y., Jiang F.L., Guo Q.L., Jiang P., Liu Y. (2024). “Multi-in-One” Yolk-Shell Structured Nanoplatform Inducing Pyroptosis and Antitumor Immune Response Through Cascade Reactions. Small.

[3] Burdette B.E., Esparza A.N., Zhu H., Wang S. (2021). Gasdermin D in pyroptosis. Acta Pharm. Sin. B.

[4] Wang Q., Wang Y., Ding J., Wang C., Zhou X., Gao W., Huang H., Shao F., Liu Z. (2020). A bioorthogonal system reveals antitumour immune function of pyroptosis. Nature.

[5] Su W., Qiu W., Li S.J., Wang S., Xie J., Yang Q.C., Xu J., Zhang J., Xu Z., Sun Z.J. (2023). A Dual-Responsive STAT3 Inhibitor Nanoprodrug Combined with Oncolytic Virus Elicits Synergistic Antitumor Immune Responses by Igniting Pyroptosis. Adv. Mater..

[6] Wang Y.Y., Wang J., Wang S., Yang Q.C., Song A., Zhang M.J., Wang W.D., Liu Y.T., Zhang J., Wang W.M. (2024). Dual-Responsive Epigenetic Inhibitor Nanoprodrug Combined with Oncolytic Virus Synergistically Boost Cancer Immunotherapy by Igniting Gasdermin E-Mediated Pyroptosis. ACS Nano.

[7] Ding B., Chen H., Tan J., Meng Q., Zheng P., Ma P., Lin J. (2023). ZIF-8 Nanoparticles Evoke Pyroptosis for High-Efficiency Cancer Immunotherapy. Angew. Chem. Int. Ed. Engl..

[8] Zhang Q., Shi D., Guo M., Zhao H., Zhao Y., Yang X. (2023). Radiofrequency-Activated Pyroptosis of Bi-Valent Gold Nanocluster for Cancer Immunotherapy. ACS Nano.

[9] Rao Z., Zhu Y., Yang P., Chen Z., Xia Y., Qiao C., Liu W., Deng H., Li J., Ning P. (2022). Pyroptosis in inflammatory diseases and cancer. Theranostics.

[10] Jiao C., Zhang H., Li H., Fu X., Lin Y., Cao C., Liu S., Liu Y., Li P. (2023). Caspase-3/GSDME mediated pyroptosis: A potential pathway for sepsis. Int. Immunopharmacol..

[11] Yao F., Jin Z., Zheng Z., Lv X., Ren L., Yang J., Chen D., Wang B., Yang W., Chen L. (2022). HDAC11 promotes both NLRP3/caspase-1/GSDMD and caspase-3/GSDME pathways causing pyroptosis via ERG in vascular endothelial cells. Cell Death Discov..

[12] He W., Xu C., Mao D., Zheng Y., Wang N., Wang M., Mao N., Wang T., Li Y. (2023). Recent advances in pyroptosis, liver disease, and traditional Chinese medicine: A review. Phytother. Res..

[13] Yuan R., Zhao W., Wang Q.Q., He J., Han S., Gao H., Feng Y., Yang S. (2021). Cucurbitacin B inhibits non-small cell lung cancer in vivo and in vitro by triggering TLR4/NLRP3/GSDMD-dependent pyroptosis. Pharmacol. Res..

[14] Long Y., Fan J., Zhou N., Liang J., Xiao C., Tong C., Wang W., Liu B. (2023). Biomimetic Prussian blue nanocomplexes for chemo-photothermal treatment of triple-negative breast cancer by enhancing ICD. Biomaterials.

[15] Feng Z., Chen G., Zhong M., Lin L., Mai Z., Tang Y., Chen G., Ma W., Li G., Yang Y. (2023). An acid-responsive MOF nanomedicine for augmented anti-tumor immunotherapy via a metal ion interference-mediated pyroptotic pathway. Biomaterials.

[16] Xu Z., Gao Y., Zhang L., Gao Y., Liao Y., Liang Y., Yuan Z., Li Y., Zhao B., Wen G. (2025). Multifunctional nanoagent for enhanced cancer radioimmunotherapy via pyroptosis and cGAS-STING activation. J. Nanobiotechnol..

[17] Huang H.T., Chang A.Q., Peng H., Liu J., Yao A.N., Ruan Y.D., Zhang P.Z., Wang T.S., Qu C.H., Yin X.B. (2024). Preparation and anti-tumor effect in hepatocellular carcinoma treatment of AS1411 aptamer-targeted polyphyllin II-loaded PLGA nanoparticles. J. Sci.-Adv. Mater. Dev..

[18] Jiao Y., Xin M., Xu J., Xiang X., Li X., Jiang J., Jia X. (2022). Polyphyllin II induced apoptosis of NSCLC cells by inhibiting autophagy through the mTOR pathway. Pharm. Biol..

[19] Miao W., Wang Z., Gao J., Ohno Y. (2024). Polyphyllin II inhibits breast cancer cell proliferation via the PI3K/Akt signaling pathway. Mol. Med. Rep..

[20] Xiao X., Zou J., Bui-Nguyen T.M., Bai P., Gao L., Liu J., Liu S., Xiao J., Chen X., Zhang X. (2012). Paris saponin II of Rhizoma Paridis--a novel inducer of apoptosis in human ovarian cancer cells. Biosci. Trends.

[21] Li J.K., Sun H.T., Jiang X.L., Chen Y.F., Zhang Z., Wang Y., Chen W.Q., Zhang Z., Sze S.C.W., Zhu P.L. (2022). Polyphyllin II Induces Protective Autophagy and Apoptosis via Inhibiting PI3K/AKT/mTOR and STAT3 Signaling in Colorectal Cancer Cells. Int. J. Mol. Sci..

[22] Messmer M.N., Snyder A.G., Oberst A. (2019). Comparing the effects of different cell death programs in tumor progression and immunotherapy. Cell Death Differ..

[23] Tang R., Xu J., Zhang B., Liu J., Liang C., Hua J., Meng Q., Yu X., Shi S. (2020). Ferroptosis, necroptosis, and pyroptosis in anticancer immunity. J. Hematol. Oncol..

[24] Aachoui Y., Sagulenko V., Miao E.A., Stacey K.J. (2013). Inflammasome-mediated pyroptotic and apoptotic cell death, and defense against infection. Curr. Opin. Microbiol..

[25] Liu Y., Zhao X., Gan F., Chen X., Deng K., Crowe S.A., Hudson G.A., Belcher M.S., Schmidt M., Astolfi M.C.T. (2024). Complete biosynthesis of QS-21 in engineered yeast. Nature.

[26] Karmakar M., Minns M., Greenberg E.N., Diaz-Aponte J., Pestonjamasp K., Johnson J.L., Rathkey J.K., Abbott D.W., Wang K., Shao F. (2020). N-GSDMD trafficking to neutrophil organelles facilitates IL-1beta release independently of plasma membrane pores and pyroptosis. Nat. Commun..

[27] Xie W., Peng M., Liu Y., Zhang B., Yi L., Long Y. (2023). Simvastatin induces pyroptosis via ROS/caspase-1/GSDMD pathway in colon cancer. Cell Commun. Signal..

[28] Liu M., Gao M., Shi X., Yin Y., Liu H., Xie R., Huang C., Zhang W., Xu S. (2024). Quercetin attenuates SiO_(2)_-induced ZBP-1-mediated PANoptosis in mouse neuronal cells via the ROS/TLR4/NF-kappab pathway. J. Environ. Manag..

[29] Stockwell B.R. (2022). Ferroptosis turns 10: Emerging mechanisms, physiological functions, and therapeutic applications. Cell.

[30] Zheng P., Ding B., Jiang Z., Xu W., Li G., Ding J., Chen X. (2021). Ultrasound-Augmented Mitochondrial Calcium Ion Overload by Calcium Nanomodulator to Induce Immunogenic Cell Death. Nano Lett..

[31] Giacomello M., Drago I., Pizzo P., Pozzan T. (2007). Mitochondrial Ca^2+^ as a key regulator of cell life and death. Cell Death Differ..

[32] Zhang X.L., Zhu J.X., Wang S.H., Li S.Y., Jiaoting E., Hu J.H., Mou R.S., Ding H., Yang P.P., Xie R. (2024). A Copper/Ferrous-Engineering Redox Homeostasis Disruptor for Cuproptosis/Ferroptosis Co-Activated Nanocatalytic Therapy in Liver Cancer. Adv. Funct. Mater..

[33] Klotz L.O., Sanchez-Ramos C., Prieto-Arroyo I., Urbanek P., Steinbrenner H., Monsalve M. (2015). Redox regulation of FoxO transcription factors. Redox. Biol..

[34] Pang K., Shi Z.D., Wei L.Y., Dong Y., Ma Y.Y., Wang W., Wang G.Y., Cao M.Y., Dong J.J., Chen Y.A. (2023). Research progress of therapeutic effects and drug resistance of immunotherapy based on PD-1/PD-L1 blockade. Drug Resist. Updates.

[35] Toldo S., Abbate A. (2024). The role of the NLRP3 inflammasome and pyroptosis in cardiovascular diseases. Nat. Rev. Cardiol..

[36] Liu Y., Zhang W., Zhou H., Chen J. (2023). Steroidal saponins PPI/CCRIS/PSV induce cell death in pancreatic cancer cell through GSDME-dependent pyroptosis. Biochem. Biophys. Res. Commun..

[37] Zhu J., Zhou W., Yao Y., Zhou X., Ma X., Zhang B., Yang Z., Tang B., Zhu H., Li N. (2024). Targeted Positron Emission Tomography-Tracked Biomimetic Codelivery Synergistically Amplifies Ferroptosis and Pyroptosis for Inducing Lung Cancer Regression and Anti-PD-L1 Immunotherapy Efficacy. ACS Nano.

[38] Chen L., Ma X., Liu W., Hu Q., Yang H. (2023). Targeting Pyroptosis through Lipopolysaccharide-Triggered Noncanonical Pathway for Safe and Efficient Cancer Immunotherapy. Nano Lett..

[39] Wu J., Liu N., Chen J., Tao Q., Lu C., Li Q., Chen X., Peng C. (2025). Clofarabine induces tumor cell apoptosis, GSDME-related pyroptosis, and CD8(+) T-cell antitumor activity via the non-canonical P53/STING pathway. J. Immunother. Cancer.

[40] Liu P., Shi X., Peng Y., Hu J., Ding J., Zhou W. (2022). Anti-PD-L1 DNAzyme Loaded Photothermal Mn^(2+)^/Fe^(3+)^ Hybrid Metal-Phenolic Networks for Cyclically Amplified Tumor Ferroptosis-Immunotherapy. Adv. Healthc. Mater..

[41] Jiang M., Chen P., Wang L., Li W., Chen B., Liu Y., Wang H., Zhao S., Ye L., He Y. (2020). cGAS-STING, an important pathway in cancer immunotherapy. J. Hematol. Oncol..

[42] Xu X., Zheng J., Liang N., Zhang X., Shabiti S., Wang Z., Yu S., Pan Z.Y., Li W., Cai L. (2024). Bioorthogonal/Ultrasound Activated Oncolytic Pyroptosis Amplifies In Situ Tumor Vaccination for Boosting Antitumor Immunity. ACS Nano.

[43] Zhaoyun L., Wang H., Yang C., Zhao X., Hui L., Song J., Ding K., Fu R. (2025). Enhancing antitumor immunity via ROS-ERS and pyroptosis-induced immunogenic cell death in multiple myeloma. J. Immunother. Cancer.

[44] Wang K.X., Cai M.R., Yin D.G., Zhu R.Y., Fu T.T., Liao S.L., Du Y.J., Kong J.H., Ni J., Yin X.B. (2023). Functional metal-organic framework nanoparticles loaded with polyphyllin I for targeted tumor therapy. J. Sci.-Adv. Mater. Dev..

[45] Jia Z., Zhao C., Wang M., Zhao X., Zhang W., Han T., Xia Q., Han Z., Lin R., Li X. (2020). Hepatotoxicity assessment of Rhizoma Paridis in adult zebrafish through proteomes and metabolome. Biomed. Pharmacother..

[46] Huang H., Fu J., Peng H., He Y., Chang A., Zhang H., Hao Y., Xu X., Li S., Zhao J. (2024). Co-delivery of polyphyllin II and IR780 PLGA nanoparticles induced pyroptosis combined with photothermal to enhance hepatocellular carcinoma immunotherapy. J. Nanobiotechnol..

