# Supplementary material for: Polyphyllin II Triggers Pyroptosis in Hepatocellular Carcinoma via Modulation of the ROS/NLRP3/Caspase-1/GSDMD Axis
Source: Antioxidants (Basel). 2026 Jan 6;15(1):75. doi: 10.3390/antiox15010075 (PMC12838060; doi:10.3390/antiox15010075)
Supplement: Supplementary file 1 [file antioxidants-15-00075-s001.zip › antioxidants-4033336-supplementary.pdf]

Supplementary Materials for

Polyphyllin II Triggers Pyroptosis in Hepatocellular Carcinoma via Modulation of the ROS/NLRP3/Caspase-1/GSDMD Axis

This PDF file includes:

Figures S1 to S7

Supporting Figures

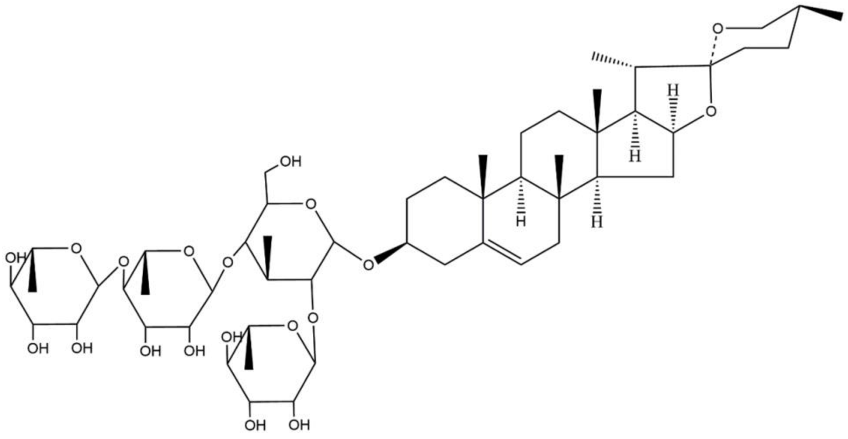

Figure S1. The chemical structure of PPII.

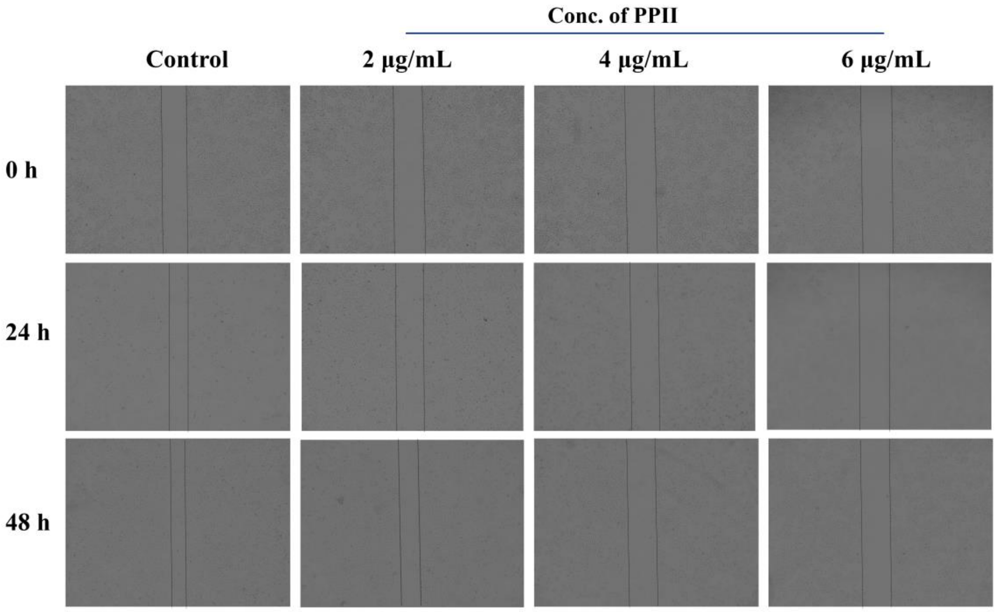

Figure S2. Representative wound healing images of HepG2 cells at 24 h and 48 h (n = 3).

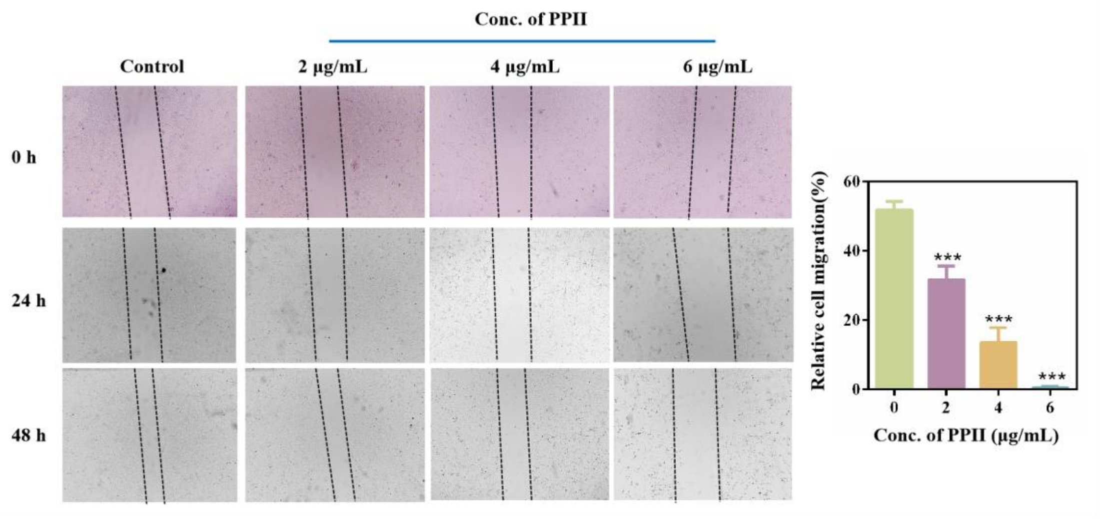

Figure S3. Representative wound healing images of Huh-7 cells at different time points and statistical analysis of migration rates at 48 h (n = 3). The p values were calculated by ANOVA with Tukey's test. ***$p < 0.001$, compared with control group.

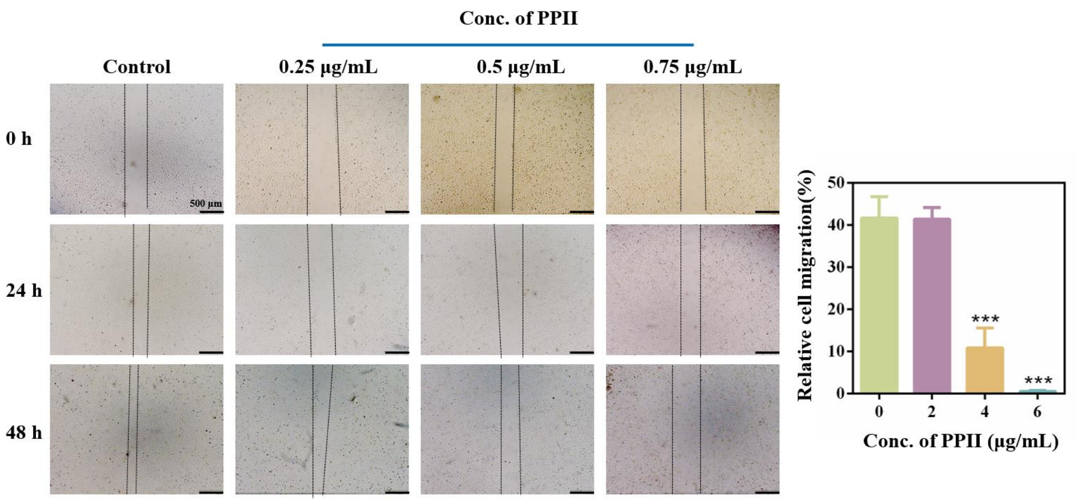

Figure S4. Representative wound healing images of SNU-449 cells at different time points and statistical analysis of migration rates at 48 h (n = 3). The p values were calculated by ANOVA with Tukey's test. ***$p < 0.001$, compared with control group.

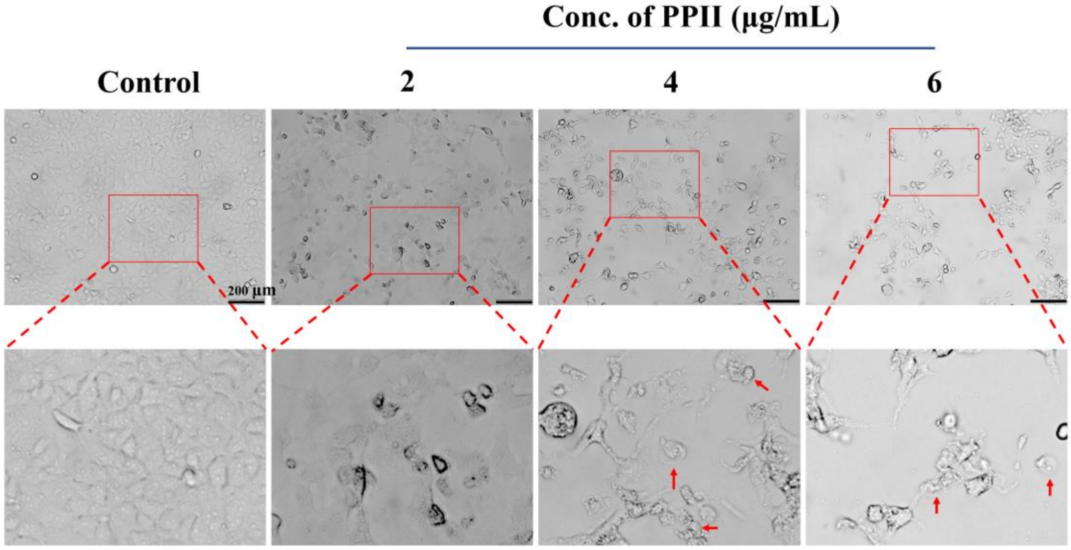

Figure S5. The morphology changes of Huh-7 cells after PPII treatment for 24 h, red arrow represents cell pyroptosis. Scale bar = 200 µm.

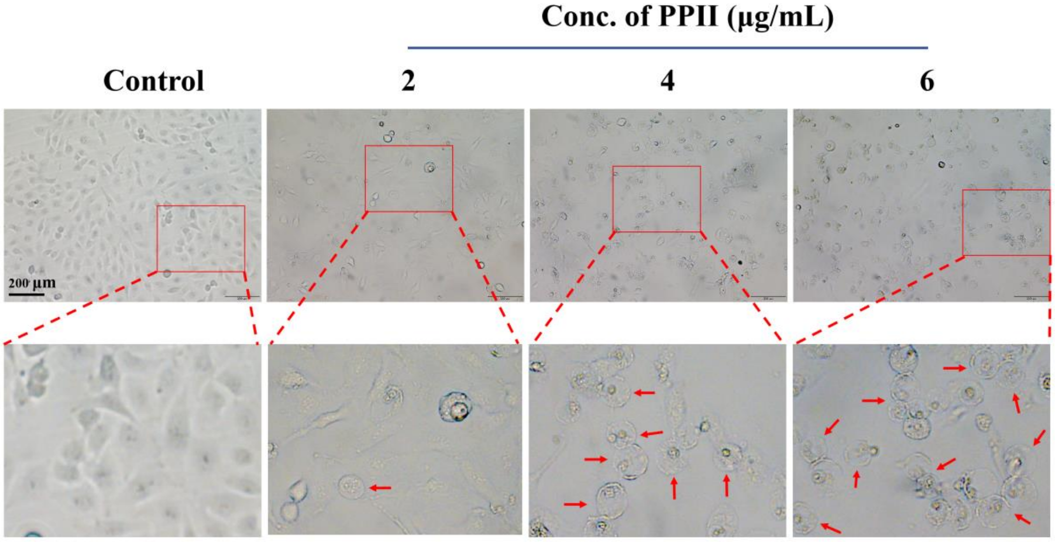

Figure S6. The morphology changes of SNU-449 cells after PPII treatment for 24 h, red arrow represents cell pyroptosis. Scale bar =200 µm.

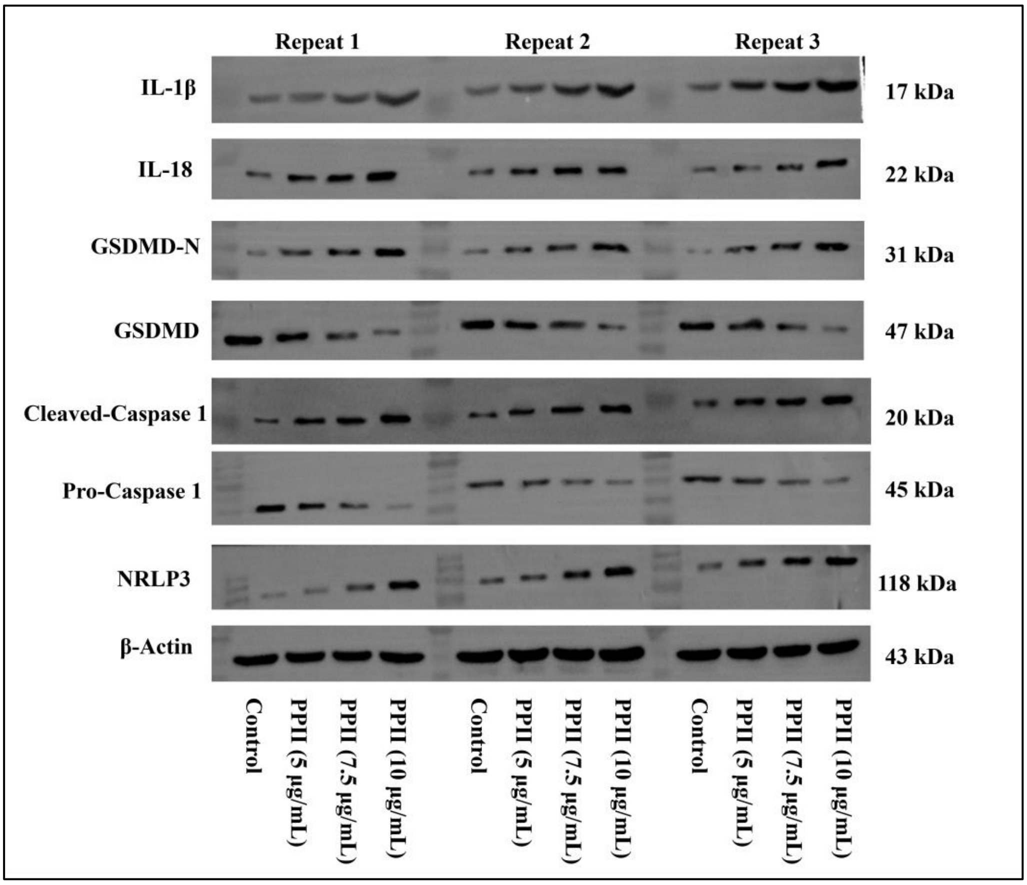

Figure S7. Original bands of IL-1β, IL-18, GSDMD-N, GSDMD, Cleaved-Caspase 1, Pro-Caspase 1, and NLRP3 proteins in tumor tissue (n = 3).
